# Supplementary material for: Chromothripsis during telomere crisis is independent of NHEJ, and consistent with a replicative origin
Source: Genome Res. 2019 May;29(5):737–49. doi: 10.1101/gr.240705.118 (PMC6499312; doi:10.1101/gr.240705.118)
Supplement: Supplemental Material [file supp_gr.240705.118_Supplemental_file_1.zip › contigs/annotated_contigs/DB47/contig.2.DB47_length_337_mean_cov_20.0830860534.docx]

**DB47_length_337_mean_cov_20.0830860534**

```
AAAAAAAAAAAAAAAAAGAAAAAAAAAAAAAAAAAAAAAAAAAAAAAAAAAAAAAAAAAAAAAAAAAAAAAAAAAAAAAAAAAAAAAAA
 >chr2:33091931-33092037 - E=2e-40 p=4e-02
AAAAAAAAAAAAAAAAA|GATATTCTGAGCCACGCATG|GTGGCTCACACCTGTAATCTCAGCACTTTGGGAGACTGAGGAGGGTGGAT
                                      >chr19:6886202-6886413 - E=9e-116
TACGAGGTCAGGAGTTCAAGACCAGCCTGGCCAAGATGGGGAAACCCCGTCTTTACTAAAAATACAAAAAATCAGCCAGTGTGGTATGG

TGTTAGGCACCTGTAATCCCAGCTACTCCGGAGGCTGAGGCAGAGAATTGCTTGAACCCAGGAGGCGGAGGC
```
